# Idiopathic hypersomnia with a video recording of a spontaneous sleep attack: A case report

**DOI:** 10.1097/MD.0000000000036782

**Published:** 2024-02-16

**Authors:** Takamasa Asaga, Kenichi Hashimoto, Yusuke Kawamura, Naoya Fujita, Motohiro Kimata, Akinori Sekizawa, Yosuke Ono, Yasuhiro Obuchi, Nanase Kobayashi, Hideto Hirasawa, Takashi Kanbayashi, Yuji Tanaka

**Affiliations:** a Department of General Medicine National Defense Medical College, Saitama, Japan; b Department of Psychiatry and Behavioral Sciences, Tokyo Medical and Dental University Graduate School, Tokyo, Japan; c Hirasawa Sleep and Mental Clinic, Saitama, Japan; d International Institute for Integrative Sleep Medicine (WPI-IIIS), University of Tsukuba, Tsukuba, Japan; e Ibaraki Prefectural Medical Center of Psychiatry, Kasama, Japan.

**Keywords:** case report, excessive daytime sleepiness, home video, idiopathic hypersomnia, narcolepsy

## Abstract

**Rationale:**

Although patients with central disorders of hypersomnolence (CDH) exhibit characteristic symptoms of hypersomnia frequently, it takes 5 to 15 years from the onset for its diagnosis due to the lack of symptom recognition. Here, we present a case of idiopathic hypersomnia (IH), a CDH, wherein early diagnosis was aided by a video footage of a spontaneous sleep attack.

**Patient concerns:**

A 21-year-old man lost consciousness while driving and experienced an accident. He had complained of excessive daytime sleepiness (EDS) over half a year. During his hospitalization for close monitoring of the loss of consciousness, an in-room surveillance camera captured a 14-minutes long spontaneous sleep attack, during which he experienced general muscle weakness and loss of consciousness without warnings or convulsions leading to a fall from the bed. There were no abnormalities in vital signs.

**Diagnoses:**

There was no significant cataplexy and less than 2 sleep-onset rapid eye movements (SOREM) in 2 sleep latency tests, with a mean sleep latency of 2.1 and 4.6 minutes. Other sleep deprivation syndromes were excluded from differential diagnosis and finally, a diagnosis of IH was confirmed according to the criteria of the Third Edition of the International Classification of Sleep Disorders. During the course of the disease, attention-deficit/hyperactive disorder (ADHD) and a gaming disorder also diagnosed.

**Interventions:**

Pharmacological treatment with modafinil was administered for IH and methylphenidate for ADHD. Cognitive behavioral therapy was performed for the gaming disorder.

**Outcomes:**

The EDS improved, and sleep attacks were no longer observed. The disruption of daily life caused by the gaming disorder was also reduced.

**Lessons:**

Video recordings of sleep attacks are beneficial for identifying the cause of loss of consciousness. Home video recordings may be helpful in the early diagnosis of IH.

## 1. Introduction

Idiopathic hypersomnia (IH) is a central disorder of hypersomnolence (CDH) characterized by excessive daytime sleepiness (EDS), prolonged nighttime sleep, long naps, and difficulty in waking up.^[1,2]^ The prevalence of IH is estimated to be 0.002% to 0.010% in the general population^[3]^ and is lower than that of narcolepsy, another common CDH. The EDS of IH is considered weaker than that of narcolepsy,^[1]^ but can lead to sleep attacks causing the patient to fall asleep in situations where they would not normally doze off, thereby leading to road traffic accidents.^[4]^ To diagnose IH, it is necessary to differentiate between reflex syncope, epilepsy, sleep apnea syndrome (SAS), periodic limb movement disorder, and drug abuse. Although differentiation is performed through detailed interviews, sleep diaries, polysomnography (PSG), multiple sleep latency tests (MSLT), and genetic testing,^[1,2]^ to our knowledge, there have been no reports on the usefulness of video recordings of sleep attacks. Here, we present a case of IH in which a video-captured spontaneous sleep attack aided in the diagnosis.

## 2. Case report

A 21-year-old male Japanese college student complaining of loss of consciousness was admitted to our hospital for examination of the same. He presented with a history of smartphone gaming disorder, and in elementary school, he tended to be forgetful, had uneven concentration, and made many careless mistakes. The patient had been experiencing repeated loss of consciousness for the past 6 months and had experienced a crush accident while driving a car 3 days earlier. The duration of the loss of consciousness was unknown. He denied the use of illicit drugs. First, a negative head-up tilt-table test was performed. Subsequently, the patient watched a video on his smartphone while eating a snack; he experienced general muscle weakness and loss of consciousness without any signs or convulsions and fell off the bed. He did not report excessive emotional reactions, such as laughing, anger, nervousness, joy, and sadness, during the video game by smartphone. The duration of the loss of consciousness was approximately 14 minutes. The patient condition was recorded on the surveillance camera in his room (Supplemental Video, http://links.lww.com/MD/L320). His attack was atypical for epilepsy with no seizures, and additional interviews revealed episodes of chronic EDS and cataplexy, leading to the suspicion of CDH. The total sleep time was <660 minutes in 24 hours. An MSLT was performed, with an average sleep latency of 2 minutes 6 seconds and 1 sleep-onset rapid eye movement (SOREM) period (Table 1). Moreover, electroencephalography showed no obvious epileptic waves. PSG revealed an apnea hypopnea index (AHI) of 0.9 and negative SAS. He was highly suspected with narcolepsy and was started on modafinil 200 mg/day. The WAIS-III test revealed an IQ of 102, with a significant group index difference of 22, and along with consideration of his clinical course, he was subsequently diagnosed with attention-deficit/hyperactive disorder (ADHD) and concomitantly treated with methylphenidate (20 mg/day). Genetic testing which was performed for auxiliary diagnosis, showed the expression of HLA-DBQ1*0301 and HLA-DBQ1*0603 and no HLA-DBQ1*0602 abnormalities typical of narcolepsy.^[5]^ A Lumbar test performed 8 months after the patient initial hospitalization, with the patient consent, showed that the orexin (hypocretin-1) concentration in the cerebrospinal fluid (CSF) was 339.6 pg/mL (cutoff value for narcolepsy: ≤110 pg/mL)^[6]^ and did not decrease. He returned to college 10 months after his first admission. However, due to his suspected CHD, he could not pursue his goal of becoming an emergency medical technician, and he became mentally unstable and discontinued his medication. Later, when he could no longer continue his schoolwork and spent more time at home, he developed a gaming disorder (ICD-11) by smartphone. His gaming debts were extremely high; he borrowed a large amount of money from consumer credit companies. Fifteen months after his initial hospitalization due to an automobile accident, he was admitted to a psychiatric department with a diagnosis of a gaming disorder by smartphone. After about a month, he was discharged from the hospital with day care and cognitive behavioral therapy. Twenty-seven months after his initial hospitalization for the automobile accident, an MSLT was performed again once the patient was off psychotropic medications, with an average sleep latency of 4 minutes 36 seconds and 1 SOREM session (Table 1). Incidentally, the PSG on the day before the MLST showed no apneas or SOREMs. The total sleep time was 511 minutes in 24 hours. As cataplexy could not be replicated and less than 2 SOREM session, the diagnosis of narcolepsy was excluded by the third edition of the International Classification of Sleep Disorders (ICSD-3) diagnostic criteria^[1]^ (ICSD-3) of narcolepsy (Supplementary Table 1–2, http://links.lww.com/MD/L318, http://links.lww.com/MD/L317). Finally, IH was diagnosed based on ICSD-3diagnostic criteria (Supplementary Table 3, http://links.lww.com/MD/L319).^[1,7]^ Currently, the patient is taking modafinil 200 mg/day as a single agent, and his daytime sleepiness is under control to the extent that it does not interfere with his daily life.

**Table 1:** Multiple sleep latency test.

	MSLT 1 (first time)					MSLT 2 (second time)				
	Nap 1	Nap 2	Nap 3	Nap 4	Nap 5	Nap 1	Nap 2	Nap 3	Nap 4	Nap 5
Starting time	9:00	11:00	13:00	15:00	17:00	9:00	11:00	13:00	15:00	17:00
Sleep latency (min)	2	1.5	0.5	3.5	3	3.0	9.5	4.5	2.5	3.5
REM sleep latency (min)	-	1	-	-	-	-	-	-	1.5	-
Average sleep latency (min)	2.1					4.6				
SOREM periods	1/5					1/5				

MSLT = multiple sleep latency test, REM = rapid eye movement, SOREM = sleep onset rapid eye movement.

## 3. Discussion

Narcolepsy type 1 was initially suspected based on a history of EDS and cataplexy. There were no other EDS-causing factors, such as sleep insufficiency, SAS, periodic limb movement disorder, delayed sleep-wake phase disorder, drugs, or similar factors.^[8]^ The diagnosis of narcolepsy type 1 is based on the presence of cataplexy, shortened sleep latency, and 2 or more SOREM periods on the MSLT or a CSF orexin level ≤ 110 pg/mL. Additional genetic testing and CSF examination were performed to confirm the diagnosis. The patient did not harbor HLA-DBQ1*0602, which is typical of narcolepsy, and his CSF orexin concentration did not decrease. In addition, the lack of reproducible cataplexy led us to suspect narcolepsy type 2. A repeat MSLT may be indicated in the following situations^[9]^:

1. When the initial test is affected by extraneous circumstances or when appropriate study conditions are not present during initial testing.
2. When ambiguous or uninterpretable findings are present.
3. When a patient is suspected of having narcolepsy and earlier MSLT evaluation(s) did not provide polygraphic confirmation.

In this case, (B) and (C) were considered the reasons for retesting. A second MSLT was performed at the right time. However, as in the first case, there was only 1 SOREM period, and IH was diagnosed. Table 2 shows the clinical characteristics of narcolepsy types 1 and 2 and IH based on the ICSD-3. IH often has a slower onset than narcolepsy, and after the onset, chronic persistent EDS is associated with a decreased quality of life.^[10]^ Narcolepsy is characterized by a short nap of approximately 10 minutes. Conversely, IH is characterized by a nap of approximately 1 hour with poor awakening and abnormal behavior called sleep drunkenness when the patient is woken by force.^[2]^ In the present case, as can be seen from the video, the patient was in a persistent drowsy state after falling out of bed and falling asleep again, a typical IH symptom. In comparison to the mean sleep latency in MSLT, the latency was reported to be 2.4 ± 1.8, 3.7 ± 2.1, and 6.2 ± 3.0 minutes for narcolepsy with and without cataplexy and IH, respectively.^[11,12]^ The mean sleep latency in the present case was 2.1 minute for the first MSLT and 4.6 minutes for the second MSLT, which was shorter than the mean sleep latency in previously reported cases of IH. The patient features were similar to those observed in narcolepsy. Therefore, the findings of mean sleep latency in the present case were not typical of IH.

**Table 2:** Clinical characteristics of narcolepsy and idiopathic hypersomnia.

	Narcolepsy type 1	Narcolepsy type 2	Idiopathic hypersomnia
Severity of daytime sleepiness^[1]^	Irrepressible	Irrepressible	Weak
Quality and quantity of Naps^[1]^	Short and refreshing	Short and refreshing	Long and unrefreshing
Cataplexy^[7]^	Yes	No	No
REM sleep abnormalities^[7]^	Yes	Yes	No
Sleep latency (min)^[7]^	8 or less	8 or less	8 or less
SOREM periods^[7]^	2 or more	2 or more	1 or less
Orexin concentration in CSF (pg/mL)^[7]^	110 or less	110 or more	110 or more
Frequency of HLA-DBQ1*0602^[20]^	Almost all	Medium	Low

CSF = cerebrospinal fluid, HLA-DBQ1 = Human Leukocyte Antigen-DBQ1, REM = rapid eye movement, SOREM = sleep onset rapid eye movement.

This case report describes spontaneous daytime sleep attacks associated with IH. There are no video reports of IH. Although it is possible to search the internet for videos of narcolepsy and IH sleep attacks, most of them involve acts performed by actors, and it is rare to find real-time videos that capture actual spontaneous attacks. This case report could be a useful educational tool for clinicians, nurses, laboratory technicians, other medical staff, and medical students. For narcolepsy, which has a larger number of reported cases, there is a video report on cataplexy.^[13]^ Moreover, for other sleep-related disorders, home videos have been reported to be useful in evaluating the disease type, severity, and treatment effect in non REM-related parasomnia.^[14]^ One area where video recording is more widely used is epilepsy, wherein long-term video electroencephalography (VEEG) has been incorporated into routine practice.^[15]^ A case report differentiating epilepsy from narcolepsy using VEEG has been published.^[16]^ Ricci et al reported that home videos may be useful for diagnosing seizure due to epilepsy.^[17]^ In their case, the video was useful in differentiating the cause of loss of consciousness from epilepsy and in making a differential diagnosis in a group with CDH. Home video recordings may be useful in the diagnosis of hypersomnolence, as in the diagnosis of epilepsy.

Hypersomnia and ADHD are commonly associated with each other. Lopez et al reported that 25% of patients with hypersomnia met the criteria for ADHD, and 22% of patients with ADHD met the criteria for hypersomnia.^[18]^ ADHD has also been reported as a risk factor for internet addictions^[19]^; these data are consistent with the course of this case.

## 4. Conclusion

We encountered a case of IH with ADHD and gaming disorder diagnosed after a road traffic accident caused by repeated blackouts, during one of which a video surveillance camera captured the seizures by chance, thus helping with the diagnosis. The findings of this case support the future use of cameras/videos in homes or hospitals for patients with syncope or suspected sleep attacks. Video recordings capturing spontaneous sleep attacks in patients with IH are rare, and the data from this case are highly significant from an educational perspective.

## Acknowledgments

We thank Editage for providing professional English editing of the manuscript.

## Author contributions

**Conceptualization:** Takamasa Asaga, Kenichi Hashimoto, Yusuke Kawamura.

**Data curation:** Takamasa Asaga, Kenichi Hashimoto, Nanase Kobayashi, Hideto Hirasawa.

**Formal analysis:** Takashi Kanbayashi.

**Investigation:** Takashi Kanbayashi.

**Project administration:** Kenichi Hashimoto.

**Supervision:** Nanase Kobayashi, Hideto Hirasawa, Takashi Kanbayashi, Yuji Tanaka.

**Visualization:** Kenichi Hashimoto.

**Writing – original draft:** Takamasa Asaga, Kenichi Hashimoto.

**Writing – review & editing:** Kenichi Hashimoto, Yusuke Kawamura, Naoya Fujita, Motohiro Kimata, Akinori Sekizawa, Yosuke Ono, Yasuhiro Obuchi, Takashi Kanbayashi, Yuji Tanaka.

## Supplementary Material








