# Supplementary material for: Idiopathic hypersomnia with a video recording of a spontaneous sleep attack: A case report
Source: Medicine (Baltimore). 2024 Feb 16;103(7):e36782. doi: 10.1097/MD.0000000000036782 (PMC10869082; doi:10.1097/MD.0000000000036782)
Supplement: Supplementary file 2 [file medi-103-e36782-s002.docx]

Supplementary Table 1

| The third edition of the International Classification of Sleep Disorders (ICSD-3) diagnostic criteria of narcolepsy type1 | |
| --- | --- |
|  | All of the following criteria must be met: |
| A | The patient has daily periods of irrepressible need to sleep or daytime lapses into sleep occurring for at least 3 months.1 |
| B | The presence of one or both of the following: |
|  | 1.Cataplexy and a mean sleep latency of 8 minutes or less and 2 or more sleep onset rapid eye movement periods (SOREMPs) on a multiple sleep latency test (MSLT).1† A SOREMP (within 15 min of sleep onset) on the preceding nocturnal polysomnogram (PSG) may replace one of the SOREMPs on the MSLT.1 If narcolepsy type 1 is strongly suspected clinically but the MSLT criteria of B1 are not met, a possible strategy is to repeat the MSLT.1 |
|  | 2.Cerebrospinal fluid (CSF) hypocretin-1 concentration, measured by immunoreactivity, is either 110 pg/mL or less, or less than one-third of mean values obtained in normal subjects with the same standardized assay.1 |
|  |  |
|  | †Sleep laboratory testing should be performed according to standard techniques, and results should be carefully interpreted in the context of the patient’s clinical history in the presence of EDS. At least 1 week of actigraphy assessment with a sleep log is strongly recommended prior to MSLT to determine factors that may bias results (eg, insufficient sleep, shift work, or other circadian rhythm disorder).1 |
|  |  |

MSLT=multiple sleep latency test, SOREMPs=sleep onset rapid eye movement periods.
