# Supplementary material for: Idiopathic hypersomnia with a video recording of a spontaneous sleep attack: A case report
Source: Medicine (Baltimore). 2024 Feb 16;103(7):e36782. doi: 10.1097/MD.0000000000036782 (PMC10869082; doi:10.1097/MD.0000000000036782)
Supplement: Supplementary file 3 [file medi-103-e36782-s003.docx]

Supplementary Table 2

| The third edition of the International Classification of Sleep Disorders (ICSD-3) diagnostic criteria of narcolepsy type2 | |  |
| --- | --- | --- |
| All of the following criteria must be met: | |  |
| A | The patient has daily periods of irrepressible need to sleep or daytime lapses into sleep occurring for at least three months. |  |
| B | A mean sleep latency of ≤8 minutes and two or more SOREMPs are found on an MSLT performed according to standard techniques. A SOREMP (within 15 minutes of sleep onset) on the preceding nocturnal PSG may replace one of the SOREMPs on the MSLT.† |  |
| C | Cataplexy is absent. |  |
| D | Either CSF orexin concentration has not been measured or CSF orexin concentration measured by immunoreactivity is either >110 pg/mL or >1/3 of mean values obtained in normal subjects with the same standardized assay. |  |
| E | The hypersomnolence and/or MSLT findings are not better explained by other causes such as insufficient sleep, obstructive sleep apnea, delayed sleep phase disorder, or the effect of medication or substances or their withdrawal. |  |
|  |  |  |
| †Sleep laboratory testing should be performed according to standard techniques, and results should be carefully interpreted in the context of the patient’s clinical history in the presence of excessive daytime sleepiness. At least 1 week of actigraphy assessment with a sleep log is strongly recommended prior to MSLT to determine factors that may bias results (eg, insufficient sleep, shift work, or other circadian rhythm disorder).1 | |  |
|  |  |  |
|  |  |  |
|  |  |  |

CSF= cerebrospinal fluid, MLST=multiple sleep latency test, PSG= polysomnogram SOREMPs=sleep onset rapid eye movement periods.
