# Supplementary material for: Idiopathic hypersomnia with a video recording of a spontaneous sleep attack: A case report
Source: Medicine (Baltimore). 2024 Feb 16;103(7):e36782. doi: 10.1097/MD.0000000000036782 (PMC10869082; doi:10.1097/MD.0000000000036782)
Supplement: Supplementary file 4 [file medi-103-e36782-s004.docx]

Supplementary Table 3

|  | The third edition of the International Classification of Sleep Disorders (ICSD-3) diagnostic criteria of idiopathic hypersomnia |
| --- | --- |
| A. | All of the following criteria must be met: |
| B. | Daily daytime sleepiness, defined as an “irrepressible need to sleep” or daytime sleep, that has been present at least 3 months |
| C. | No cataplexy |
|  | MSLT shows one of the following: |
|  | a.Fewer than 2 SOREMPs; Or |
|  | b.No SOREMPs, if the REM latency on the preceding overnight sleep study was less than or equal to 15 minutes. |
| D. | The presence of one or both of the following: |
|  | a.Average sleep latency of less than or equal to 8 minutes on MSLT; |
|  | b.Total 24-hour sleep time is greater than or equal to 660 minutes (more typically 12-14 hours) when measured in one of the following 2 ways: |
|  | 1.a 24-hour sleep study that was performed after correcting any chronic sleep deprivation; or |
|  | 2.a 24-hour sleep study that was performed after correcting any chronic sleep deprivation; or |
| E. | Insufficient sleep syndrome is ruled out (if deemed necessary, by lack of improvement of sleepiness after an adequate trial of increased nocturnal time in bed, preferably confirmed by at least a week of wrist actigraphy) |
| F. | Another condition (sleep disorder, medical or psychiatric disorder, or drug/medication use) does not better explain the hypersomnolence and test results. |

MSLT=multiple sleep latency test, PSG= polysomnogram, SOREMPs= sleep onset rapid eye movement periods.
